# Tuning Ferromagnetism in a Single Layer of Fe above Room Temperature

**DOI:** 10.3390/ma15031019

**Published:** 2022-01-28

**Authors:** Ryszard Zdyb, Krisztián Palotás, Eszter Simon, Tomasz Jaroch, Zbigniew Korczak

**Affiliations:** 1 Institute of Physics, Maria Curie-Sklodowska University, 20-031 Lublin, Poland; tomasz.jaroch@mail.umcs.pl (T.J.); stefan.korczak@mail.umcs.pl (Z.K.); 2 Wigner Research Center for Physics, Institute for Solid State Physics and Optics, Konkoly-Thege M. Str. 29-33, 1121 Budapest, Hungary; palotas.krisztian@wigner.hu; 3 MTA-SZTE Reaction Kinetics and Surface Chemistry Research Group, University of Szeged, Rerrich B. tér 1, 6720 Szeged, Hungary; 4 Department of Theoretical Physics, Budapest University of Technology and Economics, Budafoki út 8, 1111 Budapest, Hungary; eszter.simon@cup.uni-muenchen.de; 5 Department of Chemistry, Ludwig Maximilians University, 81377 Munich, Germany

**Keywords:** ultrathin ferromagnetic films, Fe, SPLEEM, DFT

## Abstract

The crystallographic and magnetic properties of an Fe monolayer (ML) grown on 2 ML Au/W(110) substrate are studied with spin-polarized low-energy electron microscopy, density functional theory, and relativistic screened Korringa–Kohn–Rostoker calculations. The single layer of iron atoms possesses hexagonal symmetry and reveals a ferromagnetic order at room temperature. We experimentally demonstrate the possibility of tuning the Curie temperature and the magnitude of magnetization of the Fe monolayer by capping with Au. Taking into account several structural models, the calculation results mostly show ferromagnetic states with enhanced magnetic moments of Fe atoms compared to their bulk value and a further increase in their value after covering with Au. The theoretically calculated Curie temperatures are in fair agreement with those obtained in the experiments. The calculations, furthermore, found evidence for the presence of frustrated isotropic Fe–Fe exchange interactions, and a discussion of the structural effects on the magnetic properties is provided herein.

## 1. Introduction

The Curie temperature and the magnitude of magnetization are among the most important properties of ferromagnetic ultrathin films, which define their usefulness in technology. It is well known that they strongly depend on the layers’ crystallographic properties, including the type of structure (fcc, hcp, etc.), crystal face, and the distance between atoms. It has also been found that both of these features depend on the surrounding nonmagnetic materials [1]. Moreover, it is known that as the thickness of the film decreases down to a single atom limit, its magnetic properties drastically change. In principle, in the monolayer case the ferromagnetic order should be suppressed at nonzero temperatures, as stated by the Mermin–Wagner theorem [2]. However, in the presence of a strong enough anisotropy, thermal fluctuations can be overcome, resulting in the appearance of a long-range order of the magnetic moments. In real systems ferromagnetic films are formed on substrates, which act as a source of anisotropy, but due to the finite-size effects, the Curie temperature becomes strongly reduced and usually is well below room temperature (RT).

A model system often used in the studies of ferromagnetism of single layers is a monolayer of Fe epitaxially grown on substrates with different symmetry, including W(110) and Au(001), which possess rectangular and square lattice, respectively. Its magnetic properties, including Curie temperature of about 210 K [3] and about 315 K [4], respectively, have been reported and confirmed in a number of papers. It has also been shown that coating with other metals changes the Curie temperature and the magnetic moments of the Fe monolayer [1,3].

In the case of single layers without coating, there are only several exceptional cases of ferromagnetic films with the Curie temperature exceeding RT. These include the recently discovered VSe2 (strictly speaking the system is built of three atomic layers) with strong magnetization above 300 K [5] and among transition metals—Fe monolayer on Au(001) [4] and on double layer Au on W(110) [6]. In the latter case, the substrate morphology strongly influences the magnetic properties of the Fe layer. The width of the tungsten terraces that are separated by monatomic steps determines the Curie temperature (*T_C_*) of 1 ML Fe as follows: on wide terraces *T_C_* clearly exceeds room temperature while on very narrow ones it is close to or even below RT [6].

In this report we explore the basic magnetic properties of a single layer of iron with hexagonally arranged atoms. We demonstrate the tuning of the Curie temperature and the magnitude of magnetization of a single layer of Fe atoms by the adsorption of Au on its top. We experimentally show that *T_C_* increases with Au coverage up to 2 ML. Simultaneously, the magnitude of magnetization significantly increases up to 1 ML of Au, then decreases. Our calculations reveal that the single layer of Fe atoms surrounded by Au can be ferromagnetically ordered at temperatures above room temperature.

## 2. Materials and Methods

### 2.1. Experiment

The experiments were performed in a spin-polarized low-energy electron microscope (SPLEEM) (ELMITEC, Clausthal-Zellerfeld, Germany) with a base pressure in the high 10^−^^11^ mbar range. The instrument is a conventional low-energy electron microscope (LEEM) equipped with a spin-polarized electron gun emitting polarized electrons and a spin manipulator that allows rotation of the polarization vector ***P*** of the incident electron beam in any desired direction [7,8,9,10]. In order to obtain a magnetic image, two images with opposite ***P*** and intensities *I*_↑_ and *I*_↓_ are taken. Both *I*_↑_ and *I*_↓_ images contain structural and magnetic information, the latter being proportional to ***P*·*M***, where ***M*** is the local sample magnetization. The structural contribution is removed by image subtraction, leaving a purely magnetic image, which is normalized by the sum of the intensities and the degree of polarization ***P*** of the incident beam, resulting in the so-called exchange asymmetry $A_{ex}=\frac{1}{P}\frac{I_{↑}-I_{↓}}{I_{↑}+I_{↓}}$. Depending upon ***M*** and ***P***, which with standard GaAs photoemission cathodes are between 20 and 30%, the time necessary to obtain an image with a good signal-to-noise ratio is in the range of hundreds of ms to several seconds. This limits a time resolution of SPLEEM.

The system is equipped with water cooled, resistively heated Au and Fe evaporators. During Au and Fe deposition, the pressure was kept below 5·10^−^^10^ mbar. The W(110) crystal was cleaned in the usual way by heating in oxygen at about 1400 K and then flashing the remaining oxygen at about 2000 K. First, two monolayers of Au were deposited at 600 K. The elevated temperature during the gold growth assures monolayer-by-monolayer growth mode. The completion and appearance of the first and second Au layers are visible as a contrast change in LEEM. Next, the Fe monolayer was deposited at about 550 K. At this temperature Fe initially forms two-dimensional islands. Strong contrast between these islands and the substrate allows precise determination of the completion of the monolayer. In addition, the second iron layer produces a strong quantum-size effect contrast. These contrast mechanisms allow for a control of total coverage, with an accuracy better than 5% in the monolayer coverage range. Finally, a gold layer with a thickness up to about 2 MLs was deposited at RT onto the Fe monolayer. Deposition of Au at RT did not cause a contrast change in the LEEM image at the energy used for imaging. Therefore, the thickness of the Au capping layer was determined from the rate calibration during Au deposition onto the bare W(110) surface, resulting in an uncertainty of the thickness of the total Au capping layer of 0.15 ML. In order to determine the Curie temperature of the Fe monolayer, asymmetry images were recorded while the sample was heated in steps by radiation from a filament placed behind the tungsten substrate. The temperature was measured in the separate experiment with an Fe-constantan thermocouple attached directly to the side of the W(110) crystal, keeping exactly the same heating conditions, time, and power, as during SPLEEM image recording [6].

### 2.2. Calculations

Theoretical calculations were carried out using density functional theory (DFT). The optimizations of the atomic structure of the various considered Au*_n_*/Fe_1_/Au_2_/W (*n* = 0,1,2, all subscripts mean the number of atomic layers) interface models (see Section 3.4 for more details) were performed by using the Vienna Ab Initio Simulation Package (VASP) [11,12] within the Perdew–Burke–Ernzerhof (PBE) parametrization [13] of the Generalized Gradient Approximation (GGA) for the exchange–correlation functional. The bottom two atomic layers of slab models in the supercell were always fixed, and the atomic positions of the other atomic layers were freely relaxed in three dimensions for the (8 × 1) supercells (having a lateral dimension of 25.40 Å × 4.49 Å) and were out-of-plane relaxed for the smaller supercells (bcc and fcc surface unit cell models containing 1 atom per atomic layer, see Section 3.4 for more details). A minimum vacuum thickness of 15 Å, perpendicular to the slabs, was applied in all cases. For the considered (8 × 1) supercells a 7 × 1 × 1 k-point sampling, and for the smaller supercells a 21 × 21 × 1 k-point sampling of the Brillouin zone, were considered. The energy cutoff for the plane-wave expansion of the single electron wave functions was set to 300 eV. The magnetocrystalline anisotropy energies (MAE) were calculated as the total energy differences between configurations having ferromagnetic Fe spins pointing to different crystallographic directions (for example, MAE*^z^* ^−^^*x*^ = *E^z^* − *E^x^*) with spin-orbit coupling included.

The optimized interlayer distances of the smaller supercell models were taken into account in the subsequent self-consistent relativistic screened Korringa–Kohn–Rostoker (SKKR) [14,15,16] calculations, whereas the large (8 × 1) supercells were excluded from further analysis due to the limitations of the complex geometry treatment within SKKR. The magnetic interface models sandwiched between the semi-infinite vacuum (at the free surface side) and metallic substrates (at the substrate side) were treated self-consistently. The local spin-density approximation of DFT, as parameterized by Vosko et al. [17], and the atomic-sphere approximation with an angular momentum cutoff of *l_max_* = 2 were used. In order to describe the magnetic interactions in the Fe monolayer in the interface models, a generalized classical Heisenberg spin model was used following the sign conventions of Ref. [18] with the following Hamiltonian: $H=-\left(\frac{1}{2}\right)\underset{ij}{\sum }s_{i}J_{ij}s_{j}+\underset{i}{\sum }s_{i}Ks_{i}$. Here, $s_{i}$ is the spin unit vector at atomic site *i*, $J_{ij}$ is the magnetic exchange coupling tensor containing the isotropic Heisenberg exchange interaction ($J_{ij}=\left(\frac{1}{3}\right)Tr\left(J_{ij}\right)$), antisymmetric Dzyaloshinsky–Moriya (DM) interaction ($\left(\frac{1}{2}\right)s_{i}\left(J_{ij}-J_{ij}^{T}\right)s_{j}=D_{ij}\left(s_{i}\times s_{j}\right)$) with $D_{ij}$ the DM vector), and traceless symmetric parts [19,20], and ***K*** is the on-site anisotropy matrix. The magnetic interaction tensors ($J_{ij}$) were determined for all pairs of atomic Fe spins up to a maximal distance of $4a_{2D}$ in terms of the relativistic torque method, based on calculating the energy costs due to the infinitesimal rotations of the spins at selected sites with respect to the ferromagnetic state oriented along different crystallographic directions [20]. Based on the DFT-parametrized spin model, the magnetic ground states of the corresponding systems were determined at zero temperature by using atomistic spin-dynamics simulations following Ref. [21]. The Curie temperature for ferromagnetic ground states was obtained from the magnetization curve $\langle M^{2}\rangle \left(T\right)$ resulting from finite temperature atomistic spin-dynamics simulations [22], a recent example using this method is reported in Ref. [23].

## 3. Results and Discussion

### 3.1. Growth and Crystallographic Structure

**Figure 1:**
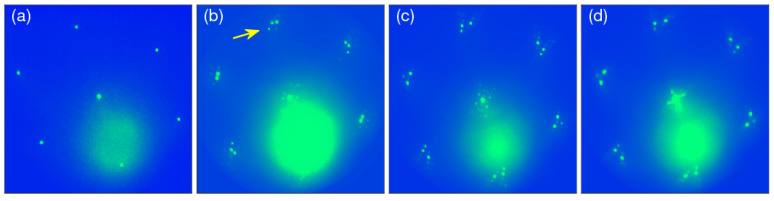
LEED patterns of bare W(110) (**a**), 2 ML Au/W(110) (**b**), 1 ML Fe/2 ML Au/W(110) (**c**), 2 ML Au/1 ML Fe/2 ML Au/W(110) (**d**). The arrow in (**b**) indicates tungsten diffraction spot. Electron energy 38 eV.

The growth conditions, the substrate surface cleanness, and its temperature during Au and Fe growth are extremely important for the evolution of the magnetic order in the Fe monolayer at RT. The criterion for the cleanness of the substrate surface was the step-flow growth of iron on the bare W(110) surface at an elevated temperature. Moreover, the Au double layer grown on the clean W(110) surface shows additional, low intensity, and sharp double scattering diffraction spots around the integer spots of the LEED pattern, Figure 1b. Small amounts of contamination, not visible in LEED, cause the appearance of many nucleation centers on the W(110) terraces during the initial stages of Fe or Au deposition. A large number of nucleation centers causes the growth of a defected layer. In the case of the Au double layer grown on a partially clean surface, there are no additional spots or they are barely visible.

The spot marked by the arrow in Figure 1b comes from the tungsten substrate, the two strong spots next to it belong to the Au double layer. They are associated with two equivalent Au domains rotated by 3.3 ± 0.4°, relative to each other. The positions of the double scattering spots are shown in a double scattering construction for the 2 ML thick Au film in Ref. [24]. The Au double layer has slightly distorted hexagonal symmetry, in which the W [001] direction approximately coincides with the Au $\left[1\bar{1}0\right]$ direction. At first glance it can be considered as a 2.9% compressed Au(111) layer with the lattice constant of 3.96 ± 0.08 Å, instead of 4.08 Å of bulk Au. Detailed analysis indicates that the distance between Au atoms along Au $\langle 11\bar{2}\rangle ,$ which is approximately parallel to W $\left[1\bar{1}0\right],$ is 4.93 ± 0.08 Å and is almost the same as in the bulk Au (5.00 Å). Along other $\langle 11\bar{2}\rangle$ directions in the Au(111) plane the corresponding distance is 4.82 ± 0.08 Å, which is about 2.2% less compared to the formerly mentioned direction (4.93 Å).

Whether the Au double layer is perfect or not is also visible during the growth of the Fe monolayer on top of Au. The growth is more random with many nucleation centers when there are no additional diffraction spots around the (00) spot in the LEED pattern. The deposition of Fe on the well-prepared Au film makes the growth more smooth and the whole 1 ML Fe/2 ML Au/W(110) system reveals double scattering spots. Within the experimental error bar, the Fe monolayer has the same crystallographic structure as the gold film below, Figure 1c. This means that the Fe monolayer has hexagonal symmetry with the lattice constant of 3.94 ± 0.08 Å corresponding to a nearest-neighbor distance of 2.79 Å. The distance between the Fe atoms along all $\langle 11\bar{2}\rangle$ directions is the same within the error bar, although the obtained values of 4.86 Å and 4.80 Å along W $\left[1\bar{1}0\right]$ and other directions, respectively, indicate a slight distortion along the W $\left[1\bar{1}0\right]$ direction. Similar to the Au double layer, the Fe monolayer is built of two domains. Interestingly, the angle between both of the domains changes to 6.8 ± 0.4°, thus, there is an obvious rearrangement in the layer morphology. The nearest-neighbor distance between the Fe atoms (2.79 Å) is much larger than the nearest-neighbor distance in bulk bcc and fcc Fe (2.48 Å and 2.54 Å, respectively), which suggests that the Fe layer is in the high spin state. The high spin state has been reported as a consequence of large lattice constant of fcc Fe [25].

It appears that the subsequent growth of Au at RT on the top of the Fe monolayer proceeds with the same crystallographic orientation, keeping the same two-domain morphology and preserving about the same lattice constant of 3.92 ± 0.08 Å and 3.93 ± 0.08 Å for 1 ML and 2 ML Au on the top of Fe, respectively, Figure 1d. The only observed change is a slight increase in the angle between the two domains, which is 7.5 ± 0.4° and 8.0 ± 0.4° for 1 ML and 2 ML Au, respectively.

### 3.2. Magnetic Structure of Fe Monolayer

**Figure 2:**
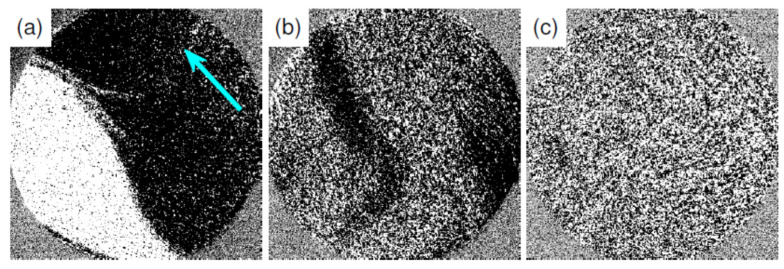
SPLEEM images of 1 ML Fe deposited on 2 ML Au/W(110) substrate recorded at RT. The Fe monolayer was deposited on the substrate with well-developed (**a**), with weak intensity (**b**) and with no (**c**) double scattering spots in LEED patterns. The arrow in (**a**) shows the tungsten $\left[1\bar{1}0\right]$ direction, which is the easy-axis of 1 ML Fe. Electron energy 3.5 eV, field of view 12 µm.

The Fe monolayer on the Au double layer that shows double scattering spots around the (00) spot is ferromagnetic, even at room temperature. Figure 2a shows the SPLEEM image of two magnetic domains with opposite magnetization directions (bright and dark areas) with an exchange asymmetry *A_ex_* of 0.008. The Fe monolayer grown on the Au double layer that shows weaker double scattering spots in the LEED pattern exhibits lower *A_ex_* values (between 0 and 0.008) and/or magnetic contrast only on a part of the surface (dark patches in Figure 2b) due to the local differences in the Curie temperature [6]. If the Au double layer shows no double scattering spots at all, then the Fe monolayer is in a paramagnetic state at room temperature, Figure 2c. The same happens when the sample temperature is too low during Fe deposition, resulting in the formation of many nucleation centers, which causes the growth of small grains. This is in agreement with the generally observed decrease of the Curie temperature with decreasing coverage and grain size—a finite-size effect [6,26,27].

The continuous Fe monolayer has uniaxial in-plane anisotropy with the easy-axis pointing in the tungsten $\left[1\bar{1}0\right]$ direction, as indicated by the arrow in Figure 2a, which approximately coincides with the $\left[11\bar{2}\right]$ direction of the Au double layer. The observed in-plane anisotropy disagrees with the reported out-of-plane anisotropy found in the Fe monolayer grown on a thick Au film [28] and in 1.5 ML Fe grown on bulk Au(111) [29,30]. It is also in contradiction to the observed out-of-plane anisotropy of Fe layers between about 0.2 and 1 ML thickness on the bulk Au(111) [31]. However, the magnetocrystalline anisotropy energy measured in the latter study was close to zero, but positive, favoring in-plane magnetization. The main difference between the present and the previous experiments can be attributed to the following different substrates: compressed Au double layer on W(110) versus Au single crystal or thick Au film [32]. It is well known that ultrathin Fe films on the bare W(110) surface have in-plane uniaxial anisotropy with the easy-axis along the tungsten $\left[1\bar{1}0\right]$ direction [33]. Our earlier experiments show that this does not change with 2 ML Au between W and Fe [32,34]. The observed distortion of the Au double layer along the W $\left[1\bar{1}0\right]$ direction can be the source of additional magnetoelastic anisotropy, which forces easy-axis along that direction. The same direction of the easy-axis was found during the RT growth of Fe on the Au double layer [32,34] in the initial coverage range. The experiments show that iron is also ferromagnetic at submonolayer coverages. One monolayer thick Fe islands reveal ferromagnetic order at room temperature with in-plane uniaxial anisotropy and magnetization along the tungsten $\left[1\bar{1}0\right]$ direction, as observed in the complete iron monolayer. Note that earlier SKKR calculations also reported an in-plane easy-axis along the W $\left[1\bar{1}0\right]$ direction for Fe ML on W(110), which, however, can be reoriented to out-of-plane [110] by changing the out-of-plane Fe atomic layer relaxation [35].

### 3.3. Magnetic Structure of Au-Capped Fe Monolayer

**Figure 3:**
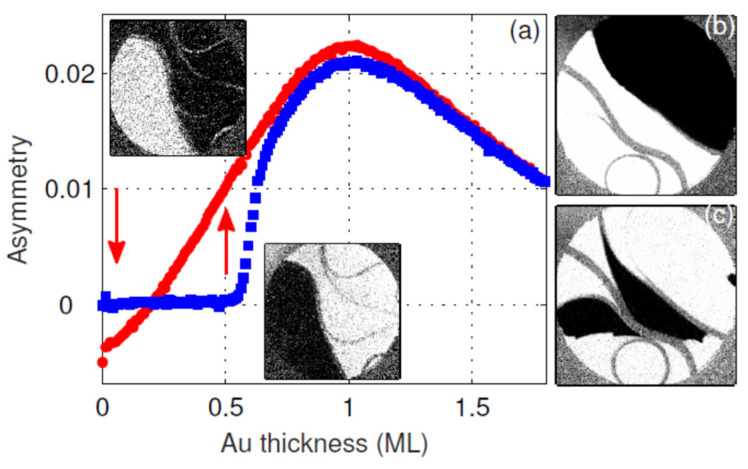
(**a**) Asymmetry vs Au capping layer coverage. The SPLEEM images in the inset were taken at Au coverages shown by the arrows. In (**b**,**c**) SPLEEM images taken before ferromagnetic-paramagnetic and after paramagnetic-ferromagnetic phase transitions, respectively. The gray features, e.g., circle, denote step bunches that are paramagnetic. Electron energy 3.5 eV, field of view 12 µm.

During the Au deposition onto the Fe monolayer the exchange asymmetry changes, as shown in Figure 3a. It starts from the value of the uncapped Fe ML, changes sign at about 0.25 ML Au, and increases approximately linearly up to about 1 ML Au. The SPLEEM images, recorded on both sides of *A_ex_* = 0, at the thicknesses indicated by the arrows in Figure 3a, show the same domain configuration but with reversed magnetization direction (reversed dark and bright regions). The contrast reversal without a change in the domain shapes, except for the small fluctuations observed occasionally at the domain boundaries during Au deposition, is reproducible in all experiments. This indicates a quantum-size effect (QSE) origin of the observed change in the sign of *A_ex_*, as previously observed in the Fe/W(110) system [36,37,38], rather than a spin reorientation transition (SRT). In SRT, the size and distribution of the magnetic domains change as observed in other studies [34,39,40,41] or as observed here upon heating and cooling during the ferromagnetic-paramagnetic-ferromagnetic phase transitions, Figure 3b,c. The observation of the magnetic images with ***P*** parallel to the W(110) [001] and [110] directions at the Fe thickness where *A_ex_* = 0 does not give magnetic contrast at all. This also confirms the QSE origin of the observed contrast reversal.

**Figure 4:**
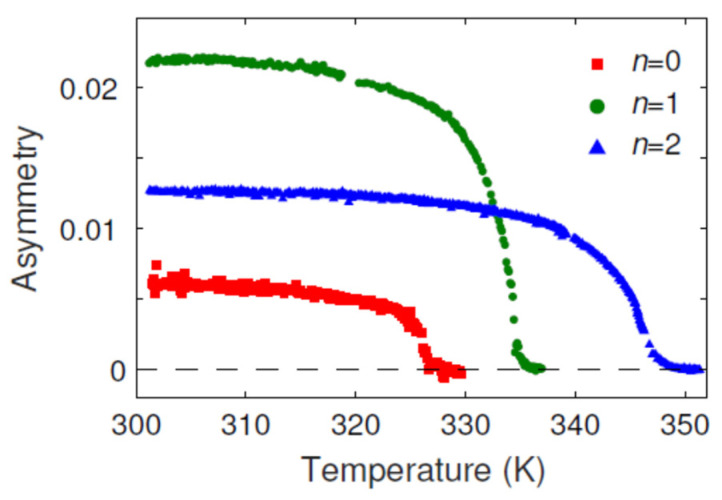
Asymmetry vs. temperature of *n* ML Au/1 ML Fe/2 ML Au/W(110).

The areas which show no magnetic contrast are often associated with the high step density of the tungsten substrate. As the terraces become narrower the Curie temperature decreases due to the finite-size effect, as discussed in detail in Ref. [6]. Apparently, the same effect causes a decrease in *Tc* in the samples that are grown at lower temperatures, at which the Fe layer does not grow in the step-flow way and, instead, it forms smaller grains. In the case of the Fe monolayer that does not show magnetic contrast at RT, the Au overlayers induce ferromagnetic order, as illustrated in Figure 3a (blue curve)—the magnetic order appears at about 0.55 ML Au. Depending on the preparation conditions of the Fe monolayer (substrate cleanness and temperature), the onset of magnetism was observed between 0 and about 0.6 ML Au. After the onset, the exchange asymmetry rapidly increases reaching at about 0.7 ML Au the same value as in the case of the samples with the magnetic contrast existing before the Au deposition starts. The sudden increase in the exchange asymmetry parameter suggests the change in the Curie temperature of the Fe film. Apparently, the addition of a certain number of Au atoms increases *Tc* above RT, resulting in the steep increase in *A_ex_*. An approximately linear increase in *A_ex_* with Au coverage of up to about 1 ML suggests its dependence on the number of Au atoms sitting on the Fe monolayer. The linear increase in *A_ex_* stops at the coverage of about 1 mL and then starts to decrease with further increasing Au coverage. Simultaneously, the Curie temperature of the Fe monolayer changes quite significantly. Without Au adsorbed on the top of Fe, it is about 327 ± 3 K, with 1 ML Au—at 335 ± 3 K and with 2 ML Au it is about 346 ± 3 K. The observed changes in the exchange asymmetry with sample temperature are shown in Figure 4.

### 3.4. Results of Calculations

**Figure 5:**
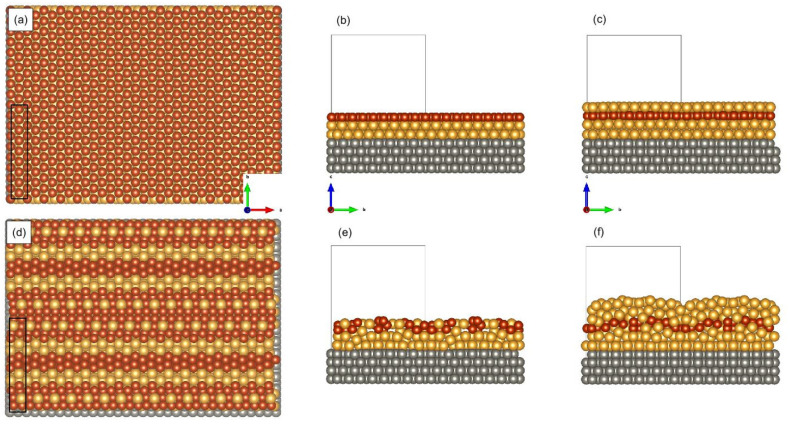
Atomic geometries of the 9:8 superstructures: (**a**) A0 model before relaxation (top view, black rectangle denotes the surface unit cell with dimension 4.49 Å × 25.40 Å; altogether 16 × 2 surface unit cells are shown with image dimension 71.84 Å × 50.80 Å), (**b**) A0 model before relaxation (side view), (**c**) A1 model after relaxation (side view), (**d**) A0 model after relaxation (top view, same dimensions as (**a**)), (**e**) A0 model after relaxation (side view), (**f**) A2 model after relaxation (side view). Atomic colors: gray (W), yellow (Au), brown (Fe). The mixing of Fe–Au atoms is evident in (**d**–**f**). Crystallographic directions: W$\left[1\bar{1}0\right]$ (red arrow), W$\left[00\bar{1}\right]$ (green arrow), W[110] (blue arrow). The supercell is indicated by the black rectangles.

We modeled the Au*_n_*/Fe_1_/Au_2_/W (*n* = 0; 1; 2) interfaces in five different ways. Models A, B, and C include a W substrate having four W, two Au, one Fe, and *n* Au atomic layers in the VASP slab optimization, and for models B and C the same structures with a semi-infinite W substrate in the SKKR calculations are taken. Models D and E have no W substrate included, and they have six Au, one Fe, and *n* Au atomic layers in the VASP slab optimization, and the same structure with a semi-infinite Au substrate in the SKKR calculations. Details on the multilayer models are summarized as follows:

**Model A**. includes 9:8 superstructure, where the bcc(110) W substrate is considered with a DFT-optimized lattice constant of 3.175 Å, and a (8 × 1) supercell is taken (containing 16 W atoms and 18 Au or Fe atoms in the corresponding atomic layers in the surface cell), where the supercell size is $3.175\times \sqrt{2}=4.49$ Å in the W $\left[1\bar{1}0\right]$ direction and 3.175 × 8 = 25.40 Å in the W [001] direction (along which nine Au atoms are for eight W, which the reason for the 9:8 superstructure), as an illustration see Figure 5a,b for *n* = 0. Here, it is assumed that the W–Au interface changes crystallographic growth type from bcc(110) for W to close to fcc(111) for Au. The in-plane nearest-neighbor Au–Au distances of the initial configurations are as follows 2.82 Å (= 25.40/9, this value is close to the experimentally determined distance along the W [001] direction), 2.75, and 2.56 Å, slightly distorted from a perfect hexagonal fcc(111) atomic layer. Above the first Au atomic layer the structure is assumed to grow epitaxially following the slightly distorted fcc(111)-type structure. This largest supercell atomic model is simplified to smaller supercell models with one atom per surface unit cell in each atomic layer with the indicated crystal structure throughout the full multilayer structure.

**Model B**. includes bcc(110) structure, including W substrate with a DFT-optimized lattice constant of 3.175 Å. This model assumes bcc(110) structure for the Au/Fe/Au parts of the multilayers.

**Model C**. includes fcc(111) structure, including W substrate with experimental in-plane lattice constant of 2.82 Å. In this model the fcc(111) structure is imposed on the W substrate.

**Model D**. includes fcc(111) structure, without W substrate with experimental in-plane lattice constant of 2.82 Å. In this model the local geometry of the Au/Fe/Au is taken from the experiment.

**Model E**. includes fcc(111) structure without W substrate with a DFT-optimized in-plane Au lattice constant of 2.953 Å.

In the following we refer to the above structural models (A–E) together with the Au capping layers (*n* = 0; 1; 2), for example, A0 refers to model A with no Au capping layer on Fe. Altogether, fifteen (5:A–E × 3:*n* = 0; 1; 2) multilayer structures were constructed, and analyzed theoretically.

After geometry optimization, for the 9:8 superstructures the expected (flat) multilayer structure was obtained for model A1 only (see Figure 5c), where one of the initial in-plane nearest-neighbor Au–Au distances was left unchanged at 2.82 Å (this value is close to the experimentally determined distance along the W [001] direction), and the other two Au–Au distances were both changed to 2.65 Å, still distorted from a perfect hexagonal fcc(111) atomic layer, but were more symmetric after the optimization. The obtained Fe spin moments are almost uniformly 2.84 µ_B_ and the induced spin moments of Au at Fe-neighboring atomic layers are smaller than 0.04 µ_B_. The MAE^*z* − *x*^ = −0.5 meV/Fe (*x* = W$\left[1\bar{1}0\right]$, *z* = W[110]), which means an out-of-plane magnetocrystalline anisotropy. Interestingly, for the A0 and A2 models we found that a mixing of Fe and Au atoms is energetically favored compared to the multilayers, and the corresponding structural models are shown in Figure 5d–f. For these models, the Fe spin moments vary in the range of 2.74–2.98 µ_B_, and the induced spin moments of Au are always smaller than 0.07 µ_B_. MAE was not calculated for these intermixed interfaces. 

In the following we focus on the smaller supercell models B-E and analyze their magnetic properties in more detail. The obtained Fe spin moments and MAE values are summarized in Table 1. The Fe spin moments vary in the range of 3.08–3.43 µ_B_, and the induced spin moments of Au at Fe-neighboring atomic layers are always smaller than 0.04 µ_B_. We observe slightly enlarged Fe spin moments for model E, where the DFT-optimized in-plane lattice constant of Au (2.953 Å) was employed, which is almost 5% larger than the experimentally observed Au–Au distance of 2.79 Å. Interestingly, for *n* = 0 the MAE results are quite insensitive to the variation of the structural model. For *n* = 1 the models B and C (with W substrate) result in larger MAE than models D and E (without W substrate).

For *n* = 2 model B shows the largest MAE in absolute value among all the considered interface models, this is clearly related to the imposed bcc(110) structure, and the fcc(111) structures (models C–E) result in the lowest MAE values. Importantly, we obtained out-of-plane magnetocrystalline anisotropy in all of the considered systems in our calculations. We cannot conclude on the total anisotropy (MAE + other terms) based on our ab initio calculations. The difference in the experimentally determined magnetic anisotropy might stem from other contributions that are present in the real samples but not captured in our ab initio modelling. Another reason could be a mismatch in the magnetic layer relaxation in comparison to the experiment. By using the SKKR method it was shown that the easy-axis can be reoriented by tuning the Fe layer relaxation in Fe/W(110) [35], clearly indicating a sensitivity of the magnetic properties on the structural model.

**Figure 6:**
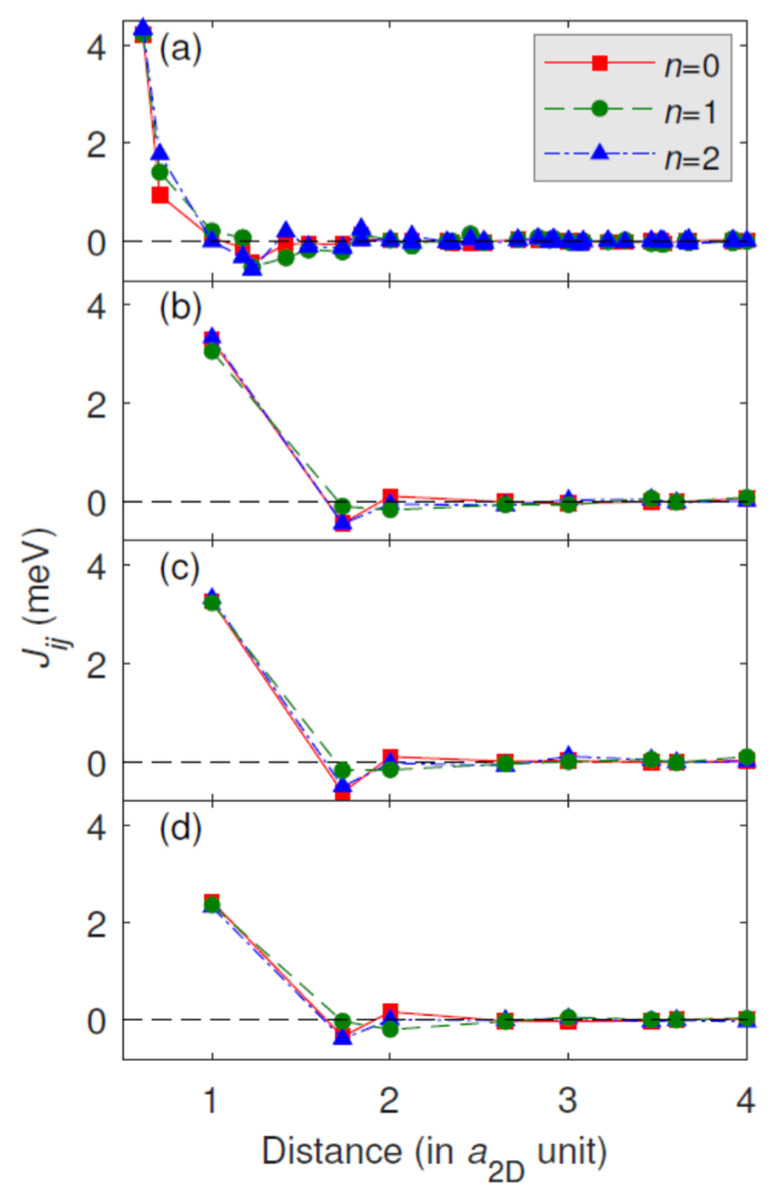
Calculated Fe–Fe isotropic exchange interaction (*J_ij_*) as a function of the interatomic distance measured in units of the in-plane lattice constant (*a*_2D_) for structural models of Au*_n_*/Fe_1_/Au_2_/W: (**a**) model B, (**b**) model C, (**c**) model D, (**d**) model E. The number of *n* Au capping layers is explicitly indicated. Here, the positive sign of *J_ij_* means ferromagnetic and the negative sign refers to antiferromagnetic coupling.

**Figure 7:**
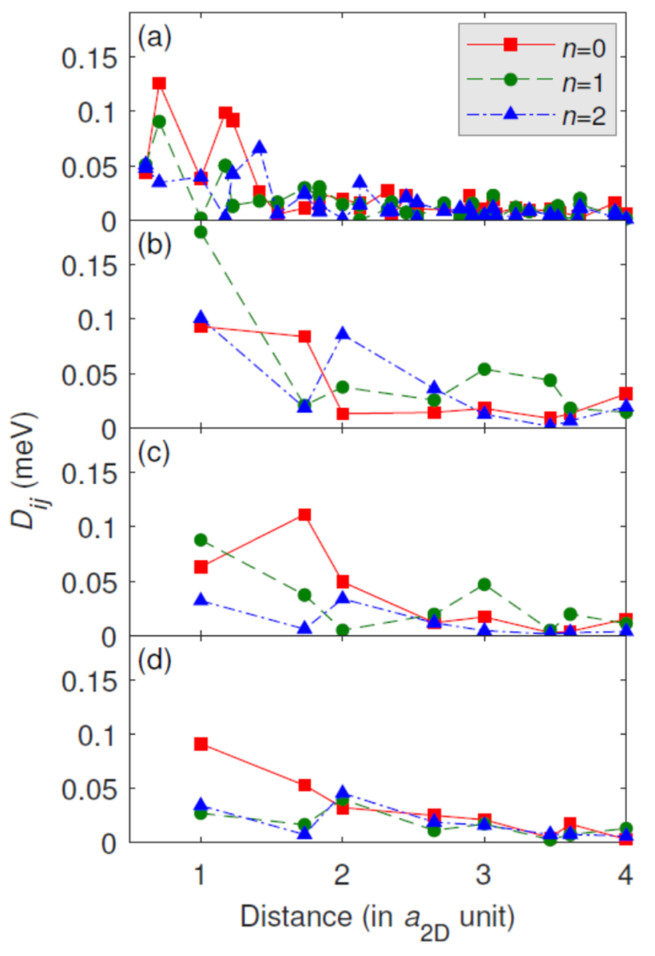
Magnitude of the Fe–Fe DM vectors (|*D_ij_*|) as a function of the interatomic distance, measured in units of the in-plane lattice constant (*a*_2D_) for structural models of Au*_n_*/Fe_1_/Au_2_/W: (**a**) model B, (**b**) model C, (**c**) model D, (**d**) model E. The number of *n* Au capping layers is explicitly indicated.

Figure 6 and Figure 7 show the calculated Fe–Fe isotropic and the magnitude of the DM interactions, respectively, for structural models B–E. The nearest-neighbor (NN) isotropic couplings are ferromagnetic for all of the multilayer systems. The second nearest-neighbor (2NN) isotropic couplings and beyond are considerably smaller than the NN isotropic couplings. The 2NN isotropic couplings are ferromagnetic for model B with the imposed bcc(110) structure only, and for the fcc(111) geometries, i.e., models C–E, the 2NN isotropic couplings are antiferromagnetic (AFM), where *n* = 1 systematically has smaller AFM couplings than *n* = 0 or *n* = 2. Beyond the third nearest-neighbors (3NN), the isotropic couplings decay rapidly, except for model B, where some of the farther neighbors have larger values than the 3NN isotropic couplings. Typically, the seemingly small changes in the 4–8 NN (for bcc) and 2–4 NN (for fcc) isotropic Fe–Fe interactions upon Au capping should be responsible for the observed variation of the magnetic properties of Fe [21] (spin moment and Curie temperature, see later text for explanation), although a quantitative correlation is difficult to establish from such complex data. For the DM interactions (Figure 7), the decay with the Fe–Fe distance is less rapid, and the farther neighbors can also play an important role when forming a complex magnetic structure through the rotation of the spins. Qualitatively similar results were obtained for Co/Pt interfaces [18,19].

**Figure 8:**
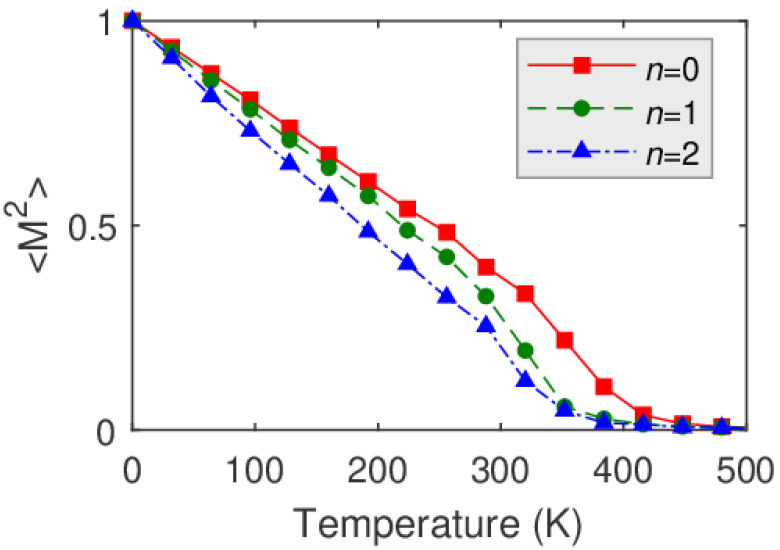
Magnetization curves $\langle M^{2}\rangle \left(T\right)$ for structural model D of Au*_n_*/Fe_1_/Au in case of *n* = 0; 1; 2.

Using the determined spin model parameters reported in Table 1 and Figure 6 and Figure 7, the magnetic ground state of Fe was calculated by atomistic spin dynamics at zero temperature. We found ferromagnetic ground states for all of the considered systems, except for models C2 and E1, where spin spiral ground states were found. For the ferromagnetic states, we calculated the magnetization curves $\langle M^{2}\rangle \left(T\right)$ resulting from finite temperature atomistic spin-dynamics simulations [22]. Examples of such magnetization curves for the structural models D are shown in Figure 8. From these, the Curie temperatures can be obtained following Ref. [23]. The results of the Curie temperatures are summarized in Table 2. Overall, the calculated Curie temperatures are fairly in the range of the experimentally determined phase transition temperatures, however, the development of the transition temperatures upon the Au addition reported in Figure 4 cannot be explained by the presented extensive model calculations. We observe oscillating (model B) or linearly changing (model D) Curie temperatures upon Au-capping depending on the structural model. We note that antiferromagnetic correlations (originated, e.g., from frustrated exchange interactions) in particular magnetic layers, tend to reduce the Curie temperature in thicker magnetic films, as was evidenced for uncapped and W-capped Fe/W(001) multilayer systems [42], an effect which might play a role here as well. We find the best quantitative agreement of the Curie temperature values for model D, where the local geometry of the Au/Fe/Au is taken from the experiment (without W substrate). Interestingly, the determined Curie temperatures of models D0 and E0 are the same, even though there is a close to 5% difference in their considered Au fcc(111) lattice constants. In general, our theoretical results also shed light on the sensitivity of the magnetic properties depending on the fine details of the underlying structural models.

## 4. Conclusions

We have investigated changes in the Curie temperature and magnitude of magnetization of 1 ML Fe grown on 2 ML Au/W(110) induced by the adsorption of Au. Spin-polarized low-energy electron microscopy experiments indicated that the Curie temperature of a single layer of iron exceeded room temperature (327 K) and it could be further increased by covering with Au atoms. In addition, capping with the gold layer initially increased the magnetic moments of the Fe atoms and, above 1 ML Au, the magnetization started to decrease. A number of applied theoretical models delivered results that qualitatively described the considered complex system, as follows: a ferromagnetic state was found for most of the structural models, also providing new physical insights, frustrated isotropic exchange interactions were identified, whereas the NN DM interaction was much smaller than its isotropic counterpart. Depending on the particular model, the Curie temperature was either close to or exceeded room temperature. The calculated magnetic moments were in the range of 3.08 to 3.43 µ_B_, clearly indicating the high spin state of the Fe atoms.

## Figures and Tables

**Table 1:** Calculated Fe spin moments (*μ_spin_* in μ_B_ units) and magnetocrystalline anisotropy energies MAE^*z* − *x*^ (*x* = W$\left[1\bar{1}0\right]$, *z* = W[110]) per Fe atom (in meV units) for the structural models B–E of Au*_n_*/Fe_1_/Au_2_/W.

*µ_spin_*/MAE^*z* − *x*^	*n* = 0	*n* = 1	*n* = 2
model B	3.09 µ_B_/−0.5 meV	3.11 µ_B_/−1.2 meV	3.21 µ_B_/−1.7 meV
model C	3.09 µ_B_/−0.5 meV	3.08 µ_B_/−1.4 meV	3.24 µ_B_/−0.4 meV
model D	3.10 µ_B_/−0.5 meV	3.10 µ_B_/−0.8 meV	3.31 µ_B_/−0.4 meV
model E	3.26 µ_B_/−0.3 meV	3.37 µ_B_/−0.9 meV	3.43 µ_B_/−0.2 meV

**Table 2:** Calculated Curie temperatures (*T_C_* in K units) for the structural models B–E of Au*_n_*/Fe_1_/Au_2_/W. SSP indicates that the ground state is not ferromagnetic, but a spin spiral is found.

*T_C_* (K)	*n* = 0	*n* = 1	*n* = 2
model B	190	450	395
model C	435	302	SSP
model D	395	360	335
model E	395	SSP	260

## Data Availability

The data are available on reasonable request from the corresponding author.
